# Aurora B maintains spherical shape of mitotic cells via simultaneously stabilizing myosin II and vimentin

**DOI:** 10.1093/jmcb/mjaf023

**Published:** 2025-08-08

**Authors:** Chenxi Hou, Fazhi Yu, Cheng Cao, Tianchen Wang, Zihang Pan, Mingru Zhong, Xing Liu, Xuebiao Yao, Kaiguang Zhang, Zhenye Yang, Jing Guo

**Affiliations:** Department of Digestive disease, The First Affiliated Hospital of USTC, Division of Life Sciences and Medicine, University of Science and Technology of China, Hefei 230027, China; MOE Key Laboratory for Cellular Dynamics, University of Science and Technology of China, Hefei 230027, China; Department of Digestive disease, The First Affiliated Hospital of USTC, Division of Life Sciences and Medicine, University of Science and Technology of China, Hefei 230027, China; MOE Key Laboratory for Cellular Dynamics, University of Science and Technology of China, Hefei 230027, China; Center for Reproduction and Genetics, Department of Obstetrics and Gynecology, The First Affiliated Hospital of USTC, Division of Life Sciences and Medicine, University of Science and Technology of China, Hefei 230001, China; MOE Key Laboratory for Cellular Dynamics, University of Science and Technology of China, Hefei 230027, China; MOE Key Laboratory for Cellular Dynamics, University of Science and Technology of China, Hefei 230027, China; MOE Key Laboratory for Cellular Dynamics, University of Science and Technology of China, Hefei 230027, China; MOE Key Laboratory for Cellular Dynamics, University of Science and Technology of China, Hefei 230027, China; MOE Key Laboratory for Cellular Dynamics, University of Science and Technology of China, Hefei 230027, China; Department of Digestive disease, The First Affiliated Hospital of USTC, Division of Life Sciences and Medicine, University of Science and Technology of China, Hefei 230027, China; Department of Digestive disease, The First Affiliated Hospital of USTC, Division of Life Sciences and Medicine, University of Science and Technology of China, Hefei 230027, China; MOE Key Laboratory for Cellular Dynamics, University of Science and Technology of China, Hefei 230027, China; Center for Advanced Interdisciplinary Science and Biomedicine of IHM, Division of Life Sciences and Medicine, University of Science and Technology of China, Hefei 230027, China; Department of Digestive disease, The First Affiliated Hospital of USTC, Division of Life Sciences and Medicine, University of Science and Technology of China, Hefei 230027, China

**Keywords:** mitosis, rounding, cortex, Aurora B, vimentin

## Abstract

Cells round up when they enter mitosis and maintain this rounded morphology until they pass the spindle assembly checkpoint during anaphase. However, the mechanisms that regulate and maintain this transient spherical state remain unclear. In this study, we demonstrate that both astral microtubules and Aurora B kinase are required to maintain cortex stability during prometaphase. Simultaneous inhibition of astral microtubules and Aurora B leads to severe and continuous deformation of mitotic cells, resulting in micronuclei containing chromosomes after the cells exit mitosis. Mechanistically, active Aurora B kinase reduces the activity of myosin light chain kinase through phosphorylation, which in turn decreases the motor activity of myosin II. Additionally, Aurora B kinase regulates the distribution of actin at the cortex by phosphorylating the intermediate filament protein vimentin. Blocking these phosphorylation events disrupts the para-cortex localization of vimentin around the cortex and leads to the dislocalization of actin at the cortex. These regulatory effects occur in highly mobile cells expressing vimentin. In summary, we show that during mitosis, Aurora B kinase coordinates the interactions between microtubules, actin, and intermediate filaments to stabilize the cortex of rounded mitotic cells, ensuring the successful completion of mitosis.

## Introduction

During mitosis, cells undergo a series of morphological changes to ensure successful cell division (Ramkumar and Baum, 2016). As cells enter mitosis, they transition into a rounded shape, which facilitates the formation of the mitotic spindle and chromosome alignment (Champion et al., 2017; Taubenberger et al., 2020). Once the mitotic checkpoint is satisfied, cells elongate and form the cleavage furrow—a contractile ring that constricts the membrane, leading to the separation of daughter cells (Kelkar et al., 2020). These dynamic changes are driven by the reorganization of the cell cortex, a thin layer of actin filaments and associated proteins beneath the plasma membrane (Dogterom and Koenderink, 2019; Svitkina, 2020). The cortex provides mechanical stability and shape to the cell, and its regulation is essential for proper cell division (Kelkar et al., 2020). Disruption of cortical stability during mitosis can result in errors in chromosome segregation, failed cytokinesis, and aneuploidy, which are characteristic features of diseases such as cancer.

The regulation of cortical instability in mitotic cells involves a complex interplay between cytoskeletal components, motor proteins, and signaling molecules. These elements work together to modulate cortical tension and shape during mitosis. The dynamics of these components are influenced by both mechanical forces and biochemical signals. Actin filaments are the fundamental building blocks of the cell cortex, and their dynamic polymerization and depolymerization drive many cortical events during mitosis. Myosin II, a motor protein, interacts with actin filaments to generate contractile forces (Kelkar et al., 2020).

During interphase, the cortex remains relatively stable, providing mechanical integrity to the cell. However, as the cell enters mitosis, the cortex undergoes extensive remodeling to facilitate the dynamic movements of chromosomes and other organelles. During mitosis, the balance between actin polymerization and depolymerization is carefully regulated by a variety of proteins. Upon entry into mitosis, the actin nucleator ARP2/3 complex is inhibited, and cyclin-dependent kinase 1 (CDK1)–cyclin B activity triggers the progressive loss of integrin-based adhesion. CDK1 coordinates the activation of ECT2, RhoA, DIA, ROCK, and myosin (Ramanathan et al., 2015), leading to the formation of a contractile actomyosin network that drives the cell into a near-spherical shape (Rosa et al., 2015; Ramkumar and Baum, 2016). Before anaphase begins, the cell must maintain this spherical and rigid state. Once cells pass the mitotic checkpoint, they elongate, and the cortex remodels to form the cleavage furrow for cytokinesis. It is well established that Aurora B kinase phosphorylates key components of the centralspindlin complex during cytokinesis to ensure proper modulation of cortical tension for cleavage furrow formation (Salbreux et al., 2012; Bovellan et al., 2014; Kim et al., 2014). However, while much is known about cytokinesis, the regulation of the stability of the rounded state during mitosis remains less well understood.

In this study, we discovered that microtubules and Aurora B kinase are essential for maintaining the rounded shape of highly motile cells during early mitosis. Disruption of astral microtubules and Aurora B kinase resulted in dramatic and constant deformation of prometaphase cells. We demonstrated that Aurora B phosphorylates myosin light chain kinase (MLCK) to prevent the over-activation of myosin II. Aurora B also phosphorylates vimentin, stabilizing it at the cortex, which is critical for maintaining the actin architecture at the cortex. These findings reveal key roles for Aurora B at the cortex during the rounding phase, in addition to its well-established functions during cytokinesis. Our data also suggest a potential strategy for targeting highly mobile cancer cells using microtubule poisons and Aurora B inhibitors.

## Results

### Concurrent disrupting astral microtubules and Aurora B induces dramatic membrane blebbing during prometaphase

To evaluate the coordination between microtubules and mitotic kinases in regulating the morphological stabilization of the mitotic cortex, we first disrupted microtubule organization using vinblastine, a clinically used drug that inhibits microtubule assembly dynamics. Next, we assessed the role of mitotic kinases in regulating cortical stability and the rounded morphology of mitotic cells. RPE1 cells were treated with CDK1 inhibitor (RO3306), Aurora A inhibitor (MLN8237), Aurora B inhibitor (Hesperadin), and Polo-like kinase 1 (Plk1) inhibitor (BI2536), either alone or in combination with vinblastine, and mitotic processes were monitored using time-lapse microscopy.

**Figure 1:**
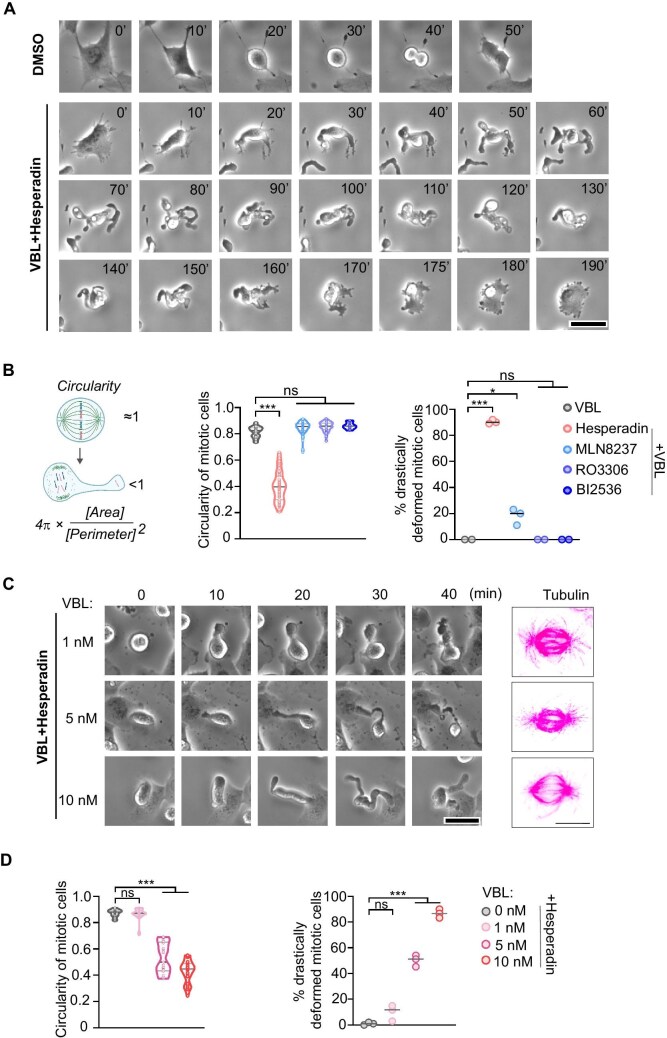
Concurrent disrupting astral microtubules and Aurora B induces dramatic membrane blebbing during prometaphase. (**A**) Live-cell imaging was performed to monitor RPE1 cell division after treatment with DMSO or 10 nM vinblastine (VBL) plus 100 nM Hesperadin. Scale bar, 20 μm. (**B**) Schematic representation of circularity measurement, along with quantification of circularity in mitotic RPE1 cells treated with 10 nM vinblastine along or in combination with 100 nM Hesperadin, 100 nM MLN8237, 10 μM RO3306, or 100 nM BI2536. A representative result from three independent experiments is shown. The proportion of drastically deformed mitotic cells (exhibiting a circularity value <0.7) is also presented, with data compiled from three independent experiments. (**C**) Left: live-cell imaging was conducted to observe RPE1 cell division under a gradient concentration of vinblastine in combination with 100 nM Hesperadin. Scale bar, 20 μm. Right: RPE1 cells were treated with increasing concentrations of vinblastine for 12 h, followed by immunofluorescence staining of β-tubulin. Representative images are shown. Scale bar, 10 μm. (**D**) Quantification of mitotic cell circularity following treatment with DMSO or increasing concentrations of vinblastine (1, 5, and 10 nM), each in combination with 100 nM Hesperadin. A representative result from three independent experiments and the proportion of drastically deformed mitotic cells obtained from three independent experiments are shown. Data are presented as mean ± SD. *P < 0.05; ***P < 0.001; ns, not significant.

Live-cell imaging revealed that the mere disruption of microtubules by vinblastine alone did not cause any distinct changes in the mitotic spherical morphology (Supplementary Figure S1A). However, when Hesperadin was used in combination with vinblastine, cells underwent dramatic morphological changes during prolonged mitosis (Figure 1A; Supplementary Videos S1 and S2). The prolonged mitosis indicated that the mitotic checkpoint remained activated due to unstable kinetochore–microtubule attachment. These cells displayed continuous deformation and blebbing until they either exited mitosis or the mitotic checkpoint was eventually satisfied (Figure 1A; Supplementary Videos S1 and S2). In contrast, mitotic cells treated with vinblastine and other kinase inhibitors did not exhibit noticeable deformation (Supplementary Figure S1A). These results suggest that the rounded morphology of mitotic cells requires Aurora B kinase activity. To quantify the roundness of the cells, we introduced the parameter circularity, which describes the shape of mitotic cells (Figure 1B). A circularity value closer to 1 indicates a more spherical shape. Consistent with the live-cell imaging data, circularity was significantly reduced in cells treated with vinblastine in combination with Aurora B inhibitor but not with other kinase inhibitors (Figure 1B). The cells exhibiting a circularity value below 0.7 are defined as dramatically deformed mitotic cells. Approximately 90% of cells under the combined treatment of vinblastine and Aurora B inhibitor displayed dramatic membrane blebbing (Figure 1B).

To investigate which components of microtubules are required for cortex stabilization, we used different concentrations of vinblastine in combination with Hesperadin. When astral microtubules were depolymerized by 5 nM vinblastine, mitotic cells still exhibited dramatic deformation in the presence of relatively normal spindle microtubules (Figure 1C and D), suggesting that astral microtubules, but not spindle microtubules, are essential for cortical stabilization. Taxol stabilizes microtubule bundles and reduces microtubule dynamics. STLC inhibits the kinesin Eg5, disrupting centrosome separation and inducing a monopolar spindle. The combination of vinblastine and either STLC or Taxol did not induce deformation of mitotic cells (Supplementary Figure S1B), further confirming that both astral microtubules and Aurora B kinase activity are required for stabilizing the mitotic rounding morphology.

### Rounding cortex disruption causes mitotic exit with whole chromosomes in micronucleus

**Figure 2:**
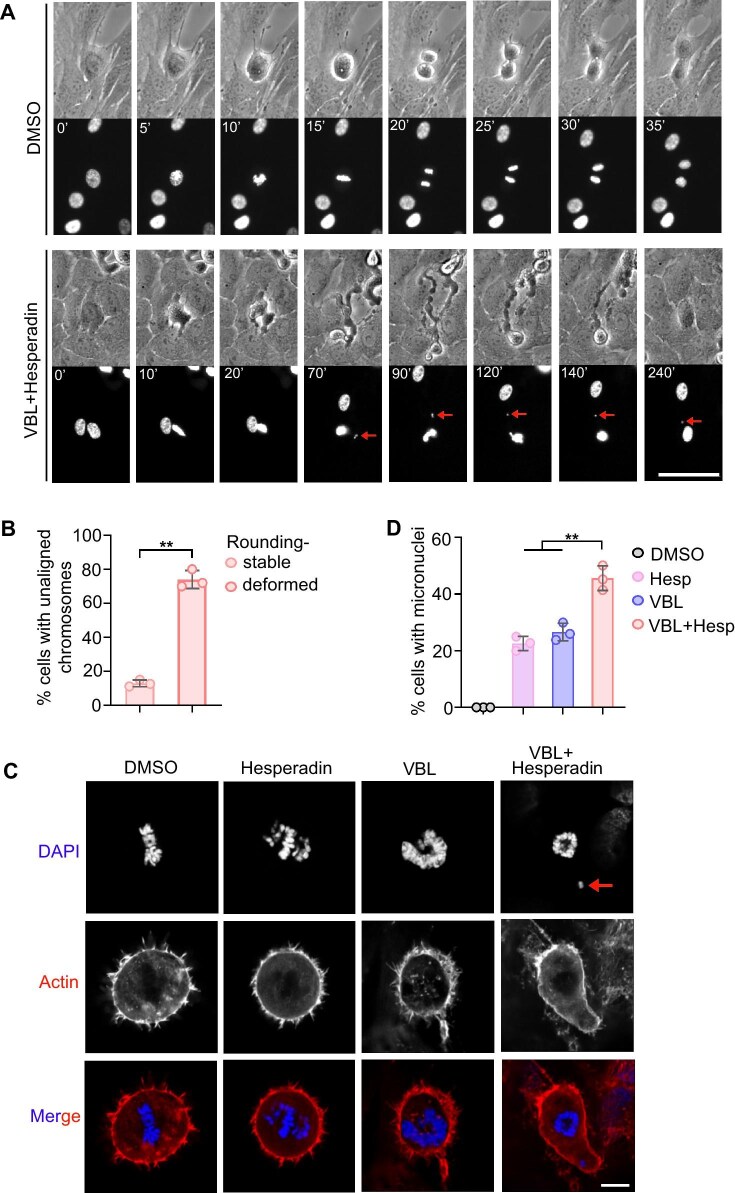
Rounding cortex disruption causes mitotic exit with whole chromosomes in micronucleus. (**A** and **B**) RPE1 cells stably expressing H2B-RFP were treated with DMSO or 10 nM vinblastine plus 100 nM Hesperadin, followed by live-cell imaging. (**A**) Representative images. Scale bar, 10 μm. Arrows indicate the detached chromosomes. (**B**) The percentage of cells exhibiting unaligned chromosomes during mitosis, categorized based on stable or deformed cell rounding. (**C** and **D**) RPE1 cells were treated with DMSO, 100 nM Hesperadin, 10 nM vinblastine, or 10 nM vinblastine plus 100 nM Hesperadin for 12 h, followed by immunofluorescence staining of actin and DAPI. (**C**) Representative images. Scale bar, 10 μm. The arrow indicates the detached chromosome. (**D**) The percentage of cells with micronuclei. Data were obtained from three independent experiments. Data are presented as mean ± SD. **P < 0.01.

To examine the impact of severe cortical deformation on cell division, we performed live-cell imaging in RPE1 cells with chromosomes labeled by H2B-GFP. When cells were treated with vinblastine and Hesperadin, they underwent severe blebbing and stretching, causing the chromosomes to become distanced from the main chromosome mass (Figure 2A). Furthermore, cells that underwent deformed rounding were more likely to produce unaligned chromosomes compared to cells with stable rounding during mitosis (Figure 2B). These unaligned chromosomes eventually formed cytoplasmic micronuclei as the cells exited mitosis (Figure 2A, arrow). Additionally, immunofluorescence analysis revealed that these micronuclei contained chromosomes (Figure 2C, arrow), which may contribute to chromosome instability and thus hinder tumor proliferation. The percentage of cells with micronuclei was significantly increased under the combined treatment of vinblastine and the Aurora B inhibitor (Figure 2D). These results suggest that the morphological deformation induced by vinblastine and Hesperadin not only disrupts the cell division process but also leads to the formation of micronuclei, further promoting chromosome instability.

### Asymmetric redistribution of actomyosin leads to morphological deformation following Aurora B and astral microtubule inhibition in mitotic cells

**Figure 3:**
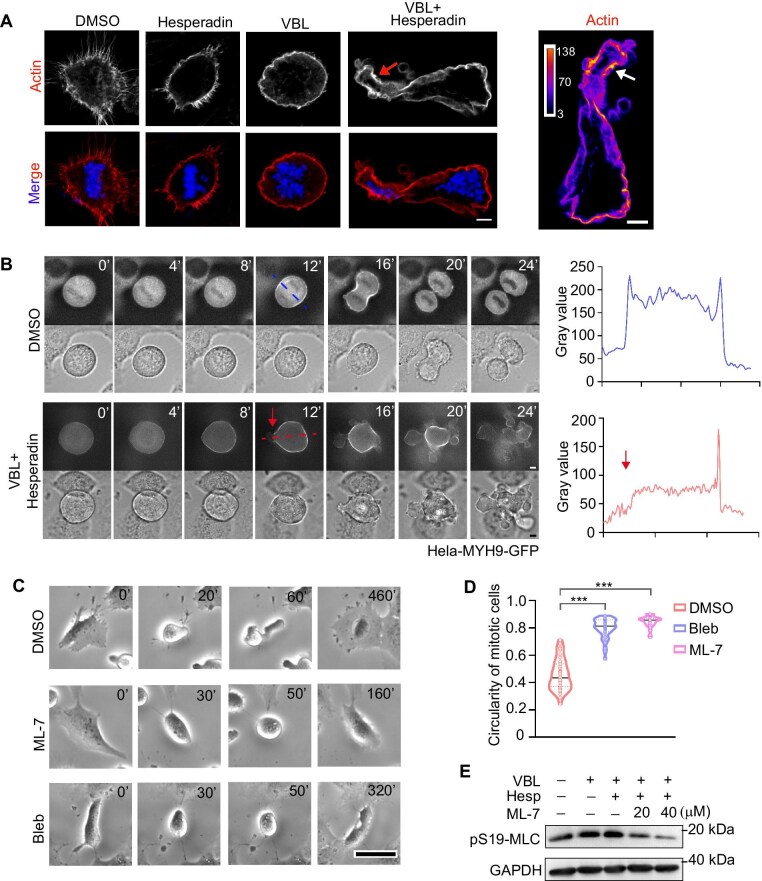
Asymmetric redistribution of actomyosin leads to morphological deformation following Aurora B and astral microtubule inhibition in mitotic cells. (**A**) RPE1 cells were treated with DMSO, 100 nM Hesperadin, 10 nM vinblastine, or 10 nM vinblastine plus 100 nM Hesperadin for 12 h, followed by immunofluorescence staining of actin and DAPI. The percentage of mitotic cells exhibiting drastically deformed morphology was quantified. Scale bar, 10 μm. (**B**) HeLa Kyoto cells stably expressing MYH9-GFP were treated with DMSO or 10 nM vinblastine plus 100 nM Hesperadin, followed by live-cell imaging. Scale bar, 10 μm. Line scan intensity of deformed positions is quantified. (**C** and **D**) RPE1 cells were treated with 10 nM vinblastine plus 100 nM Hesperadin, in the presence of DMSO, 20 μM ML-7, or 50 μM Blebbistatin (Bleb). (**C**) Representative images from live-cell imaging. Scale bar, 20 μm. (**D**) Quantification of mitotic cell circularity based on morphological observations. A representative result from three independent experiments is shown. (**E**) RPE1 cells were treated as indicated for 24 h, followed by western blot analysis of pS19-MLC levels. A representative result from three independent experiments is shown. Data are presented as mean ± SD. ***P < 0.001.

To explore how mitotic cell deformation is triggered after treatment with vinblastine and Hesperadin, we first investigated the distribution of the cortical actin network. While Hesperadin or vinblastine alone did not affect actin distribution at the cortex, the combination of these two compounds resulted in asymmetric localization of actin in the deformed mitotic cells (Figure 3A). In addition to blebbing, actin was enriched at the stretched membrane (Figure 3A, arrows). Next, we used a cell line stably expressing GFP-tagged myosin heavy chain MYH9 (Liu et al., 2015) and performed live-cell imaging to track the dynamic localization of myosin in the deformed mitotic cells (Figure 3B). In dimethyl sulfoxide (DMSO)-treated mitotic cells, MYH9 levels were low and evenly distributed in the rounded cells prior to anaphase onset. Once the cells passed the checkpoint and entered anaphase, MYH9 accumulated at the cleavage furrow, mediating abscission (Figure 3B, also shown with line scan). However, after treatment with both vinblastine and Hesperadin, MYH9 was recruited and accumulated, though it was not evenly distributed at the cortex where deformation occurred (Figure 3B, also shown with line scan). We observed a significant increase in myosin levels, which became asymmetrically distributed as deformation and blebbing began (Figure 3B, arrow). These findings suggest that inhibition of Aurora B and astral microtubules leads to asymmetric distribution of actin and myosin at the cortex, disrupting the isotropic actomyosin network and inducing pronounced deformation of mitotic cells.

When we inhibited myosin activity using Blebbistatin, the deformation of mitotic rounding was abolished (Figure 3C and D), indicating that myosin motor activity is essential for cell shape deformation during prometaphase. MLC phosphorylation serves as a biomarker for the motor activity of myosin II, while the phosphorylation of MLCK indicates inactivation of MLCK (Taneja et al., 2021). When cells were treated with the MLCK inhibitor ML-7, the deformation phenotype in mitotic cells was no longer observed (Figure 3C and D), and western blot analysis confirmed that the phosphorylation of MLCK (pS19-MLC) was inhibited by the addition of ML-7 (Figure 3E). These results suggest that inactivation of MLCK kinase activity prevents myosin activation, thereby inhibiting the deformation induced by Aurora B inhibition.

### Aurora B regulates actin distribution and myosin activity by phosphorylating vimentin and MLCK, respectively

**Figure 4:**
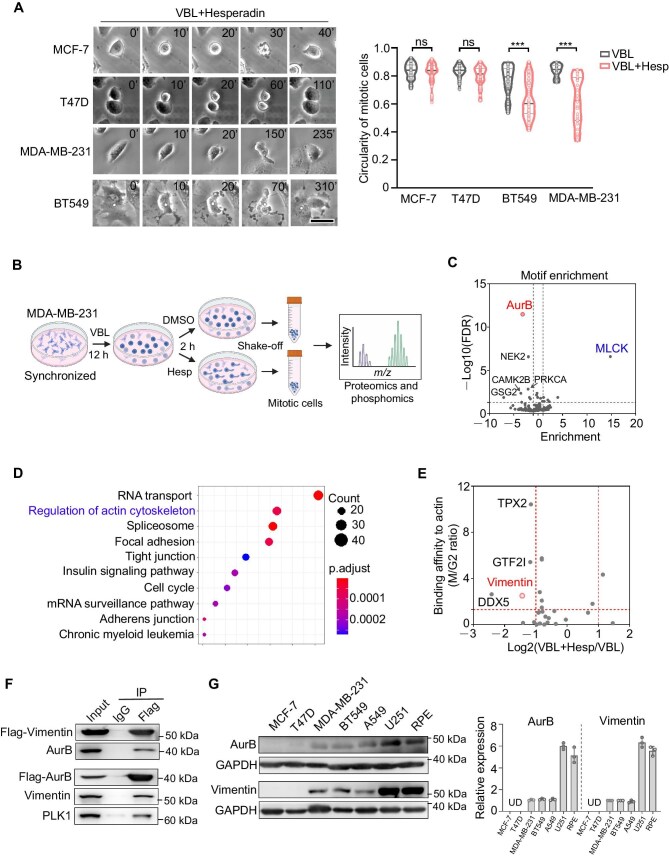
Aurora B regulates actin distribution and myosin activity by phosphorylating vimentin and MLCK, respectively. (**A**) Live-cell imaging of mitotic morphology in MCF-7, T47D, MDA-MB-231, and BT-549 cells following treatment with 10 nM vinblastine plus 100 nM Hesperadin. Scale bar, 20 μm. Mitotic cell circularity was quantified based on morphological observations. A representative result from three independent experiments is shown. (**B**) Flowchart illustrating the procedure for mitotic cell collection for proteomic and phosphoproteomic analyses. (**C**) Scatter plot displaying the downregulated enriched motifs (enrichment value <0) and upregulated enriched motifs (enrichment value >0) following Aurora B inhibition during mitosis, highlighting the shift in phosphorylation levels, particularly for Aurora B target motifs and MLCK target motifs. (**D**) KEGG pathway enrichment analysis of proteins exhibiting reduced phosphorylation upon Hesperadin treatment. (**E**) Scatter plot displaying proteins with higher actin-binding affinity during mitosis compared to interphase (M/G2), as reported in a previous study (Serres et al., 2020). The x-axis represents the log2 value of phosphorylation ratio of the combined vinblastine and Hesperadin treatment relative to vinblastine alone (VBL + Hesp/VBL). The y-axis represents the ratio of M/G2 binding affinity of actin. (**F**) 293T cells were transfected with either Flag-Aurora B or Flag-Vimentin and then treated with 10 nM vinblastine to arrest the cells in mitosis, followed by immunoprecipitation and immunoblotting. A representative result from three independent experiments is shown. (**G**) Western blot analysis of vimentin and Aurora B (AurB) protein levels across different cell lines. Data are presented as mean ± SD. ***P < 0.001; ns, not significant.

To investigate the regulation of cortex stabilization in different cell types, we used a series of breast cancer cell lines and treated them with vinblastine and Hesperadin. Among the cell lines tested, MDA-MB-231 (Supplementary Videos S3 and S4), BT549, A549, and U251 exhibited dramatic cortical deformation after cell rounding in prometaphase (Figure 4A; Supplementary Figure 2A). In contrast, MCF-7 and T47D cells maintained a spherical morphology even after astral microtubule depolymerization and Aurora B kinase inhibition (Figure 4A), suggesting that cortex stability is differentially regulated in these cell lines.

To identify the downstream targets of Aurora B substrates at the cortex, we performed phosphoproteomic analysis on MDA-MB-231 cells, with or without Aurora B inhibition (Figure 4B). First, we analyzed the motif enrichment of peptides whose phosphorylation levels were altered following Aurora B inhibition. The most downregulated enriched motif was the Aurora B target motif, while the most upregulated enriched motif was the MLCK target motif. These findings confirm that inhibition of Aurora B kinase activity upregulates MLCK kinase activity, validating the experimental approach (Figure 4C).

Next, we aimed to identify the primary pathways affected by Aurora B inhibition. Kyoto Encyclopedia of Genes and Genomes (KEGG) analysis of the proteins whose phosphorylation levels were downregulated upon Aurora B inhibition revealed that the regulation of the actin cytoskeleton was one of the most significantly altered pathways (Figure 4D). To further explore this, we integrated phosphoproteomic data (Figure 4B) with the F-actin interactome previously defined in mitotic and interphase cells using pull-down assays and mass spectrometry (Serres et al., 2020). By cross-referencing proteins that interact with F-actin and are potential Aurora B substrates, we identified vimentin as a candidate of interest (Figure 4E), given its well-established role in regulating dynamic changes in cell shape. Furthermore, reciprocal immunoprecipitation assays confirmed that Aurora B indeed interacts with vimentin during mitosis (Figure 4F).

We then examined the expression of vimentin and Aurora B kinase in these cell lines (Figure 4G). Interestingly, MCF-7 and T47D cells that did not exhibit deformation during mitosis following vinblastine and Hesperadin treatment showed very low expression levels of both vimentin and Aurora B, while cell lines that showed mitotic deformation had higher expression levels of vimentin (Figure 4G; Supplementary Figure 2B), suggesting that vimentin expression correlates with the stability of the mitotic cortex. These data indicate that high levels of Aurora B regulate vimentin distribution to stabilize the cortex during prometaphase, thereby facilitating chromosome segregation in highly mobile cells.

### Vimentin interaction controls actomyosin distribution and rounding morphology of mitotic cells

**Figure 5:**
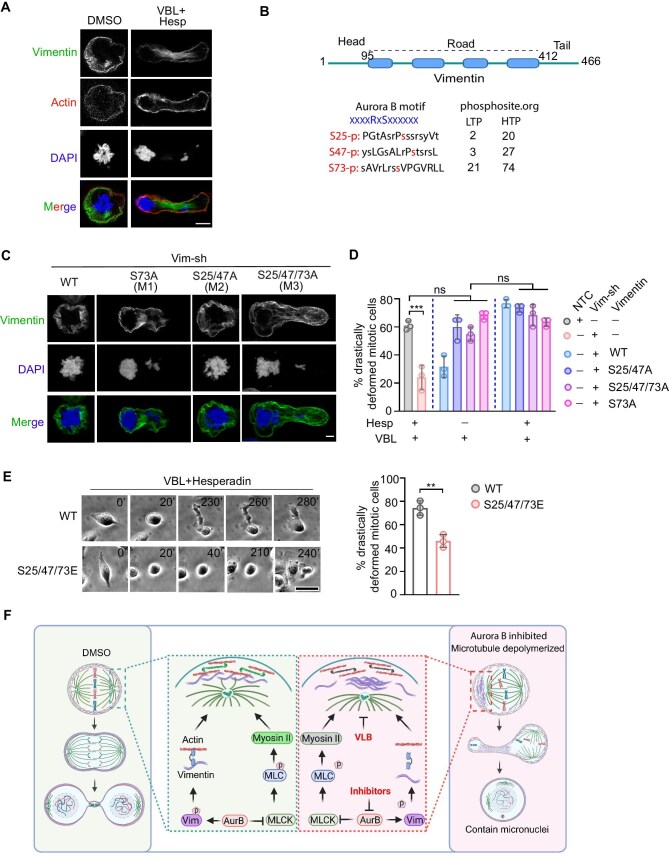
Vimentin interaction controls actomyosin distribution and rounding morphology of mitotic cells. (**A**) MDA-MB-231 cells were treated with DMSO or 10 nM vinblastine plus 100 nM Hesperadin for 12 h, followed by immunofluorescence staining of actin, vimentin, and DAPI. Scale bar, 10 μm. (**B**) A schematic representation of vimentin is presented, highlighting the phosphorylation sites targeted by Aurora B during mitosis. These phosphorylation sites were selected based on our mass spectrometry data, earlier reports (Goto et al., 2003), and predictions from the PhosphoSitePlus database regarding Aurora B motif (Kettenbach et al., 2011). LTP (low throughput papers) refers to the number of records in which this modification site was identified using methods other than discovery mass spectrometry. HTP (high throughput papers) indicates the number of records in which this modification site was assigned solely through proteomic discovery mass spectrometry. (**C**–**E**) MDA-MB-231 cells with vimentin knockdown (Vim-sh) were transfected with either wild-type (WT) or mutant vimentin expression plasmids as indicated. (**C**) Stable cell lines were treated with 10 nM vinblastine plus 100 nM Hesperadin for 12 h, followed by immunofluorescence staining to assess vimentin distribution and chromosome segregation. Scale bar, 10 μm. (**D**) The percentage of drastically deformed mitotic cells was quantified based on live-cell imaging of MDA-MB-231 stable cell lines treated with 10 nM vinblastine with or without 100 nM Hesperadin. (**E**) Live-cell imaging was performed to monitor MDA-MB-231 cell division following treatment with 10 nM vinblastine plus 100 nM Hesperadin. Scale bar, 20 μm. The percentage of mitotic cells displaying severely deformed morphology was quantified. Data were obtained from three independent experiments. Data are presented as mean ± SD. **P < 0.01; ***P < 0.001; ns, not significant. (**F**) Proposed model summarizing the findings in this study, created in BioRender.

Next, we sought to determine whether vimentin phosphorylation is involved in regulating the cell rounding morphology of mitotic cells following Aurora B and astral microtubule inhibition. First, we investigated the localization and assembly of vimentin fibers. We found that Aurora B inhibition disrupted the subcortical localization of vimentin, causing uneven accumulation of vimentin bundles without altering the expression levels (Figure 5A; Supplementary Figure S2C). Since the N-terminal internal disordered region is essential for vimentin localization (Duarte et al., 2019), we analyzed vimentin phosphorylation in this region and observed a significant decrease in phosphorylation following Aurora B inhibition in mitotic cells. We also considered the phosphorylation sites previously reported (Goto et al., 2003) and predicted by the PhosphoSitePlus database as potential Aurora B targets in mitotic cells. Based on these sources, we selected three phosphorylation sites that were most significantly altered and generated vimentin mutants, in which the corresponding serines were replaced with alanines (Figure 5B; Supplementary Figure S3A). We found that when serine 73 was mutated (S73A), vimentin formed bundles (Figure 5C). Dramatic mitotic cell deformation was observed in S73A-expressing cells following vinblastine treatment (Figure 5D), confirming that Aurora B-mediated phosphorylation regulates vimentin fiber assembly and distribution. This process is critical for actin distribution and the stabilization of cortical morphology.

Furthermore, we generated a phosphomimetic vimentin mutant in which the three serines were replaced with glutamic acid residues (S25/47/73E) and assessed whether this mutant could rescue the deformation phenotype (Figure 5E). Indeed, expression of the phosphomimetic mutant restored normal mitotic morphology (Figure 5E), indicating that phosphorylation at these sites is functionally important. Taken together, these results demonstrate that vimentin phosphorylation downstream of Aurora B is required for proper filament assembly and cortical stabilization, thereby regulating cell shape during mitosis.

## Discussion

Mitotic rounding is a dynamic process that involves the regulation of cell surface tension, actin dynamics, and interactions between membrane-associated proteins (Taubenberger et al., 2020). The stiffness, tension, and architecture of the cortex are tightly controlled by signaling networks involving actin and myosin filaments, membrane-associated proteins, and regulatory enzymes. However, the mechanisms by which cells maintain their rounded morphology during mitosis remain unclear.

In this study, we demonstrate that cells with high motility typically exhibit elevated expression of vimentin and myosin motors. Upon mitotic entry, enhanced Aurora B kinase activity plays a crucial role in maintaining the stable, rounded cell morphology observed during prometaphase and metaphase through two parallel pathways. Firstly, Aurora B phosphorylates MLCK, resulting in the inhibition of its kinase activity, which is essential for myosin activation. Secondly, Aurora B phosphorylates vimentin at serine 73, thereby preventing its assembly into bundles. This inhibition is critical for maintaining a uniform cortical actin distribution. Additionally, the anchoring of astral microtubules at the cell cortex, in conjunction with the cortical actin network, further reinforces cortex stability (Figure 5F). Collectively, our findings highlight that Aurora B-mediated phosphorylation of myosin II and vimentin serves opposing roles during prometaphase and cytokinesis concerning myosin II activity regulation. These phosphorylation events activate myosin II at the contractile ring during cytokinesis, while they suppress cortical myosin II activity during prometaphase and metaphase. Thus, our study elucidates the spatial and temporal regulation mechanisms employed by Aurora B kinase to precisely control mitotic cell morphology.

The regulation of cortex instability is crucial for proper spindle formation, chromosome congression, and cytokinesis, ensuring accurate cell division. During mitosis, local fluctuations of the cortex are modulated by various proteins that respond to cellular signaling pathways and mechanical cues. The interactions between actin and myosin are influenced by several regulatory pathways, including Rho GTPases, which control the activity of actin-binding proteins and myosin motor proteins. For example, RhoA activates Rho-associated protein kinase (ROCK), which phosphorylates MLC, enhancing myosin II activity (Champion et al., 2017; Chugh et al., 2017). This increased actomyosin contractility is a key driver of cortex instability during mitosis, particularly in the formation of the cleavage furrow during cytokinesis. The temporal and spatial regulation of Aurora B is also essential for coordinating mitotic progression with changes in cell shape. It is well established that Aurora B at the mid-zone is critical for cleavage furrow formation during anaphase and cytokinesis by coordinating myosin distribution and activation. Aurora B has also been reported to localize to the cortex during early mitosis (Dulyaninova and Bresnick, 2004; Abdullah et al., 2005). In this study, we demonstrate that Aurora B regulates both myosin II activity and the actin architecture of the cortex during mitosis. Thus, unlike its role in inducing cortex remodeling at anaphase, Aurora B stabilizes the cortex architecture during prometa- and metaphase (Supplementary Figure S3B).

While the Rho/ROCK pathway is a well-established activator of myosin II, growing evidence suggests that Aurora B can also modulate the Rho/ROCK axis in various cellular contexts, particularly during cytokinesis (Hu et al., 2008). Several studies have shown that Aurora B can phosphorylate or otherwise regulate upstream components of the Rho GTPase signaling cascade, thereby influencing myosin II activation through ROCK-dependent phosphorylation of MLC (Babkoff et al., 2021). However, in our own experiments, we did not observe significant deformation when cells were treated with the CDK1 inhibitor RO3306 or the Plk1 inhibitor BI2536 (Supplementary Figure S1A). Given that ROCK is traditionally considered a major regulator of myosin II, our findings suggest that the cell-deformation phenotype observed under Aurora B inhibition may occur partly or entirely via a ROCK-independent pathway. Thus, while Aurora B has the potential to impact the Rho/ROCK axis, our data indicate that additional or alternative mechanisms beyond classical Rho/ROCK signaling may contribute to Aurora B-dependent myosin II regulation (Supplementary Figure S3B).

Aurora B is well recognized for its central role in cytokinesis, particularly during anaphase, where it accumulates in the mid-zone to regulate cleavage furrow ingression and the final stages of cell division (Kim et al., 2014; Hadders and Lens, 2022). By coordinating the spatial activation of myosin II and the formation of the contractile ring, Aurora B helps ensure proper partitioning of chromosomes and other cellular components (Dulyaninova et al., 2004). However, previous work has focused largely on Aurora B's functions after chromosome segregation has commenced; its influence on cortical stability and shape maintenance during earlier mitotic phases remains less well characterized. Our findings complement these studies by establishing that Aurora B's regulatory role extends beyond furrow formation. Specifically, we show that Aurora B phosphorylates both MLCK and vimentin during prometaphase and metaphase, thus maintaining a uniform actomyosin cortex even before the onset of anaphase. This reveals a distinct mechanism wherein Aurora B, typically known for driving cortical remodeling in later stages, supports cortex stability in the earlier stages of mitosis (Supplementary Figure S3B).

The interaction between vimentin and actin is essential for the distribution of the actin network at the cortex (Duarte et al., 2019; Serres et al., 2020; Vadnjal et al., 2022). Vimentin undergoes conformational changes and post-translational modifications. In addition to Aurora B, CDK1 and Plk1 also phosphorylate vimentin at multiple residues (Yamaguchi et al., 2005), suggesting that vimentin is extensively phosphorylated and undergoes complex regulation. These modifications are important for its assembly and distribution. While Aurora B has been demonstrated to participate in filament breakdown in the context of anaphase cortex remodeling in some studies, our current work highlights how Aurora B-mediated phosphorylation of vimentin at serine 73 specifically limits bundle formation during earlier mitotic phases. Together, these results suggest that vimentin phosphorylation by multiple kinases constitutes an intricate system to temporally control cytoskeletal organization throughout mitosis. Although our study does not provide direct experimental evidence showing how vimentin regulates actin distribution at the molecular level, our conclusions are supported by previous findings (Duarte et al., 2019; Serres et al., 2020; Vadnjal et al., 2022) indicating that intermediate filaments can influence actin organization. Further work is required to elucidate the precise mechanisms through which vimentin exerts these effects on cortical actin.

Moreover, it is important to recognize that microtubules, actin filaments, and intermediate filaments often exist in a complex, highly integrated network (Etienne-Manneville, 2018; Dogterom and Koenderink, 2019). Aurora B's ability to modulate both vimentin and myosin activity suggests a possible coordination between the three major cytoskeletal components (Spiliotis, 2018; Nishimura et al., 2019). In addition to preventing excessive actomyosin activity, Aurora B may also inhibit actomyosin-driven membrane protrusions while reinforcing overall membrane stiffness through its downstream effects on vimentin and microtubules (Jiu et al., 2017). A similar concept underlies the observed synergistic effects of combined microtubule and Aurora B inhibition; disrupting microtubules can destabilize cortical anchoring, and inhibiting Aurora B can further diminish vimentin-dependent reinforcement of the membrane, resulting in significant cortical instability. Other regulatory factors, such as crosslinkers, motor proteins, and scaffolding proteins, that facilitate vimentin–microtubule and actin–microtubule interactions may also participate in this process and warrant additional investigation (Guan et al., 2023).

In cells with low levels of vimentin, such as HeLa and MCF-7, the rounding morphology remains stable even when both Aurora B and astral microtubules are inhibited. Although blebs occur, the overall shape does not significantly change or deform. These data suggest that isotropic actomyosin networks are more stable in these cells. In cells with high motility, microtubules, actin, and intermediate filaments all contribute to cortex stability. In contrast, in less motility cells, vimentin is absent and astral microtubule seems dispensable for maintaining the mitotic rounding.

Tumor cells undergoing epithelial–mesenchymal transition (EMT) induce vimentin expression, making their cell division more vulnerable to changes in cell shape. Our study suggests that combining Aurora B inhibitors with vinblastine could be a potential therapeutic strategy for tumor cells with high levels of vimentin or cells undergoing EMT. The induced micronuclei may activate the cGAS-mediated immune response, which could be leveraged in combination with immune checkpoint-targeting therapies. Given the implications for cell division and the potential for errors leading to diseases like cancer, further research on the regulation of cortex instability offers valuable insights into the fundamental mechanisms of cellular division and their potential as therapeutic targets.

## Materials and methods

### Cell lines and reagents

The MCF-7, T-47D, MDA-MB-231, BT-549, A549, HeLa, and HEK293T cell lines were obtained from the American Type Culture Collection (ATCC), while the U-251 MG cell line was acquired from Merck. The HeLa Kyoto cell line (characterized by high mobility) expressing MYH9-GFP was generously provided by Dr Yanjun Liu (Liu et al., 2015). All cell lines were cultured in Dulbecco's Modified Eagle Medium (DMEM; Invitrogen/Thermo Fisher Scientific) supplemented with 10% fetal bovine serum (FBS; Gibco) and 1% penicillin/streptomycin (P/S; Beyotime Biotechnology). Cells were maintained at 37°C in a humidified incubator with 5% CO₂. Routine mycoplasma testing was conducted to ensure the integrity of cell cultures.

MLN8237, Hesperadin, BI2536, vinblastine, ML-7, (−)-Blebbistatin, Taxol, and STLC were purchased from Sigma or Selleck Chemicals LLC and dissolved in DMSO. The following antibodies were used in this study: Vimentin (D21H3) XP^®^ Rabbit mAb (Cell Signaling Technology, #5741), Phospho-Myosin Light Chain 2 (Ser19) Antibody (Cell Signaling Technology, #3671), GAPDH Monoclonal Antibody (Proteintech, #60004-1-Ig), Beta Tubulin Monoclonal Antibody (Proteintech, #66240-1-Ig), and Beta Actin Monoclonal Antibody (Proteintech, #66009-1-Ig). Secondary antibodies, including HRP-conjugated goat anti-rabbit or mouse, Cy5-conjugated donkey anti-rabbit or mouse, Alexa 594-conjugated goat anti-rabbit or mouse, FITC-conjugated goat anti-human, and Alexa 488-conjugated goat anti-rabbit or mouse, were obtained from Jackson ImmunoResearch. Additionally, Alexa Fluor™ 594 (#A12381) was purchased from Thermo Fisher Scientific.

### Live-cell imaging

For live-cell imaging, 2 × 10⁴ cells were seeded into 8-well sliders (#80826, ibidi) and incubated overnight at 37°C in DMEM supplemented with 10% FBS and 1% P/S. The culture medium was then replaced with phenol red-free L-15 medium (Invitrogen/Thermo Fisher Scientific) containing 10% FBS and 1% P/S. Drugs or DMSO were added prior to imaging. Time-lapse images were captured every 5 min using a 20× objective lens on an Eclipse Ti microscope (Nikon). The duration of mitosis and interphase was determined based on morphology, with rounded-up cells categorized as mitotic and flattened cells as interphase.

### Cell synchronization

MDA-MB-231 cells were synchronized using a double thymidine block (2 mM, Sigma-Aldrich). Cells were plated in 10-cm dishes one day before treatment. Thymidine (2 mM) was added for 19 h to arrest cells, followed by three phosphate-buffered saline (PBS) washes and release into fresh medium for 9 h. A second thymidine block was then applied for 16 h, after which cells were washed three times with PBS and released into DMEM containing 10 nM vinblastine. Twelve hours later, DMSO or 100 nM Hesperadin was added to the respective groups. After an additional 2 h, mitotic cells were collected by shake-off and prepared for proteomics and phosphoproteomics analyses.

### Proteomics and phosphoproteomics

Proteomics and phosphoproteomics analyses were conducted by PTM Biolabs Inc. (Hangzhou, China). Briefly, samples were lysed with a buffer containing urea, protease, and phosphatase inhibitors. After centrifugation, protein concentration was measured using a BCA kit. Proteins were digested with trypsin after reduction and alkylation steps. Peptides were enriched by incubating with IMAC resin, washed, and eluted using ammonia solution, followed by desalting with C18 ZipTips. Peptides were separated by UHPLC and analyzed using an Orbitrap Exploris™ 480 mass spectrometer. Data-dependent acquisition mode was used to select and fragment parent ions for analysis. Detailed protocols are available from PTM Biolabs Inc.

### Western blot analysis

Cells were seeded into culture dishes 12 h before treatment. After aspirating the culture medium, cells were washed three times with PBS before lysis using the buffer (50 mM Tris–HCl, pH 7.4, 250 mM NaCl, 1 mM EDTA, 50 mM NaF, 0.5% Triton X-100, and protease inhibitors). After 30 min on ice, lysates were centrifuged at 12000 rpm for 15 min at 4°C, and supernatants were collected. Protein concentrations were determined using the Bradford assay (Sangon Biotech). Proteins were separated by SDS–PAGE, transferred onto polyvinylidene difluoride membranes (Millipore), and detected using Western Lightning Chemiluminescence Reagent Plus (Advansta).

### Immunoprecipitation

Cells were first lysed in NETN buffer (50 mM Tris–, HCl, pH 8.0, 150 mM NaCl, 0.2% nonidet P-40, and 2 mM EDTA) containing a protease inhibitor cocktail (Roche) for 20 min at 4°C. The lysate was then centrifuged at 14 000× *g* for 15 min at 4°C, and the supernatant was collected as the protein sample. For immunoprecipitation, ∼500 μg of protein was incubated with either control or specific antibodies (1–2 μg) for 12 h at 4°C under constant rotation. Next, 50 μl of 50% protein G magnetic bead slurry (Thermo Fisher) was added, and the mixture was incubated for an additional 2 h at 4°C. The beads were subsequently washed five times with the same lysis buffer, with each wash followed by magnetic stand collection (Thermo Fisher) at 4°C. Finally, the immune complexes were eluted from the beads by resuspending them in 2× SDS–PAGE loading buffer and boiling for 5 min. The boiled samples were then separated by SDS–PAGE and analyzed via immunoblotting with the appropriate antibodies.

### Gene knockdown by shRNA

Lentiviral particles were produced in HEK293T cells. Virus-containing supernatants were collected 48 h post-transfection and filtered through 0.45-μm non-pyrogenic filters (Merck Millipore). For transduction, cells were seeded at 1 × 10⁵–3 × 10⁵ cells per well in 35-mm dishes. After 24 h, lentivirus and 8 μg/ml polybrene (Sigma-Aldrich) were added. The medium was replaced 24 h later to remove viral particles. Stable cell lines were selected by adding 1 μg/ml puromycin 48 h post-infection.

### Immunofluorescence

Cells were seeded onto 22 × 22 mm coverslips 24 h prior to treatment. For tubulin staining, cells were washed with pre-warmed PHEM buffer (60 mM PIPES, pH 6.8, 25 mM HEPES, 10 mM EGTA, and 2 mM MgCl₂) and fixed with 4% formaldehyde for 15 min at room temperature. Cells were then permeabilized with 0.2% Triton X-100 in PHEM buffer, blocked with 1% bovine serum albumin in Tris-buffered saline with Tween 20 (TBST) for 30 min, incubated with primary antibodies for 2 h at room temperature, washed with TBST three times, and incubated with secondary antibodies for 1 h. For vimentin staining, cells were extracted with 0.2% Triton X-100 in PHEM buffer for 45 sec before fixation. DNA was counterstained with 4′,6-diamidino-2-phenylindole (DAPI) for 3 min. Coverslips were mounted using ProLong Antifade (Sigma). Images were captured using a high-resolution confocal microscope (LSM980, Leica).

### Statistical analysis

All statistical analyses were performed using GraphPad Prism software. Statistical significance was determined using two-way analysis of variance (ANOVA) for tumor growth analysis, log-rank tests for survival data, and two-tailed *t*-tests for other comparisons. *P* < 0.05 was considered statistically significant.

## Supplementary Material

mjaf023_Supplemental_Files
