# Supplementary material for: Aurora B maintains spherical shape of mitotic cells via simultaneously stabilizing myosin II and vimentin
Source: J Mol Cell Biol. 2025 Aug 8;17(5):mjaf023. doi: 10.1093/jmcb/mjaf023 (PMC12750452; doi:10.1093/jmcb/mjaf023)
Supplement: mjaf023_Supplemental_Files [file mjaf023_supplemental_files.zip › JMCB-2025-0073.R2_Supplementary material.pdf]

Supplementary material

Supplementary Figures

Figure S1

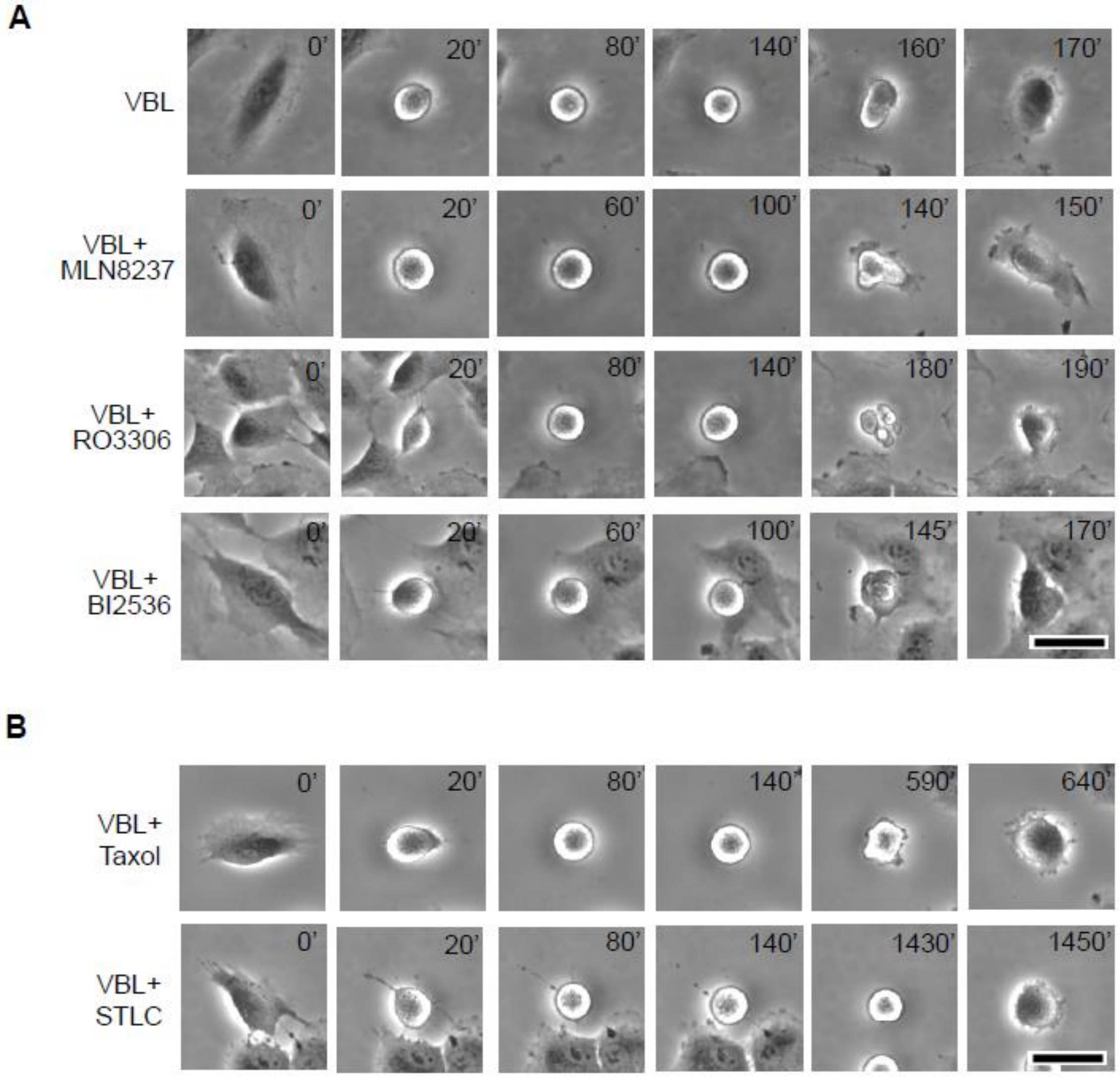

Supplementary Figure S1. Concurrent disrupting astral microtubules and Aurora B induces dramatic membrane blebbing during prometaphase. (**A**) Live-cell imaging was conducted to monitor RPE1 cells following treatment with 10 nM vinblastine (VBL) alone or in combination with 100 nM MLN8237, 10 μM RO3306, or 100 nM BI2536 (scale bar = 20 μm). (**B**) Live-cell imaging was performed to observe the division of RPE1 cells following treatment with 10 nM vinblastine in combination with Taxol or 20 μM STLC (scale bar = 20 μm).

Figure S2

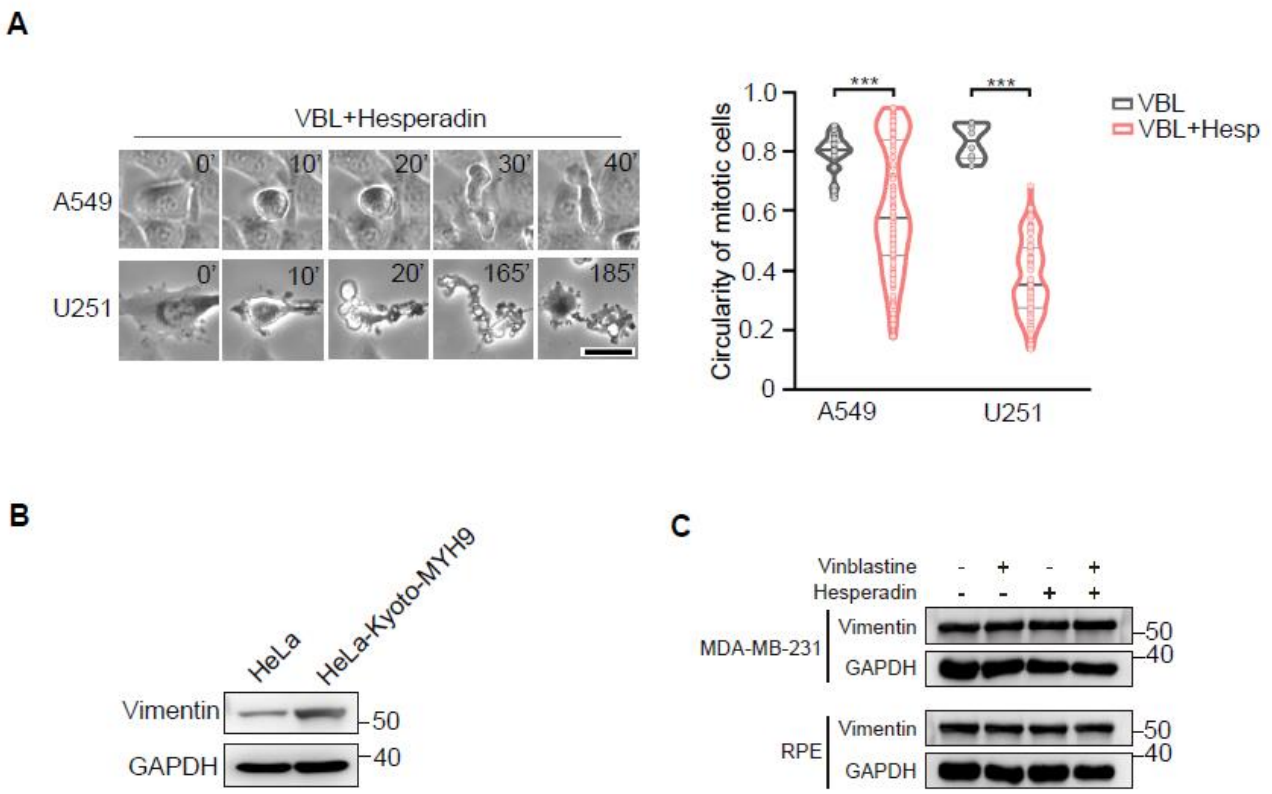

Supplementary Figure S2. (**A**) Live-cell imaging of mitotic morphology in A549 and U251 cells following treatment with 10 nM vinblastine plus 100 nM hesperadin (scale bar = 20 μm). Quantification of mitotic cell circularity based on morphological observations. Data were obtained from three independent experiments. (**B**) Western blot analysis of vimentin protein levels in HeLa and HeLa-Kyo-MYH9 cells. (**C**) Western blot analysis of vimentin protein levels in MDA-MB-231 and RPE1 cells. Cells were treated with DMSO, 10 nM vinblastine, 100 nM hesperadin, or a combination of 10 nM vinblastine plus 100 nM hesperadin.

Figure S3

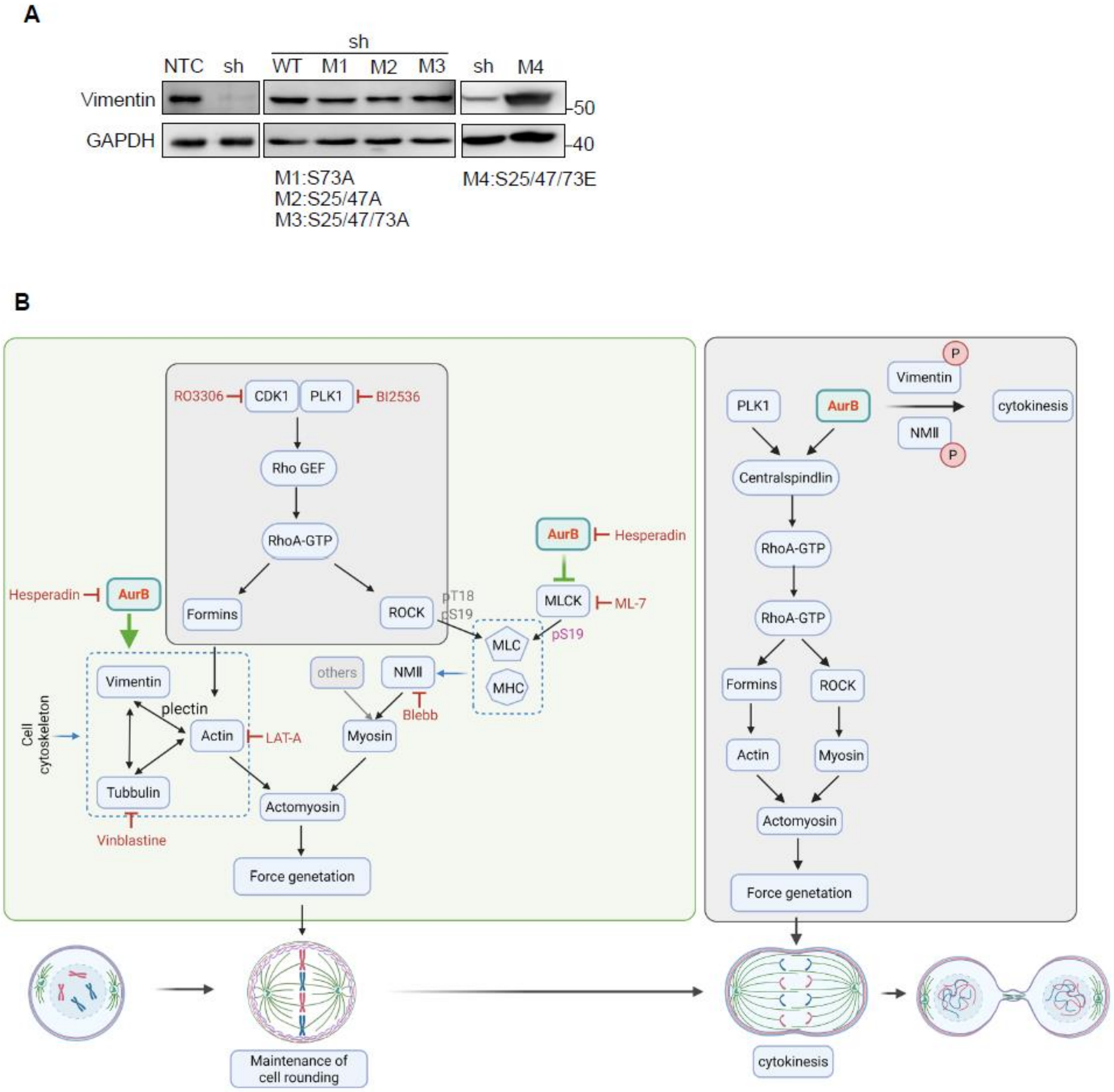

Supplementary Figure S3. (**A**) Western blot analysis of vimentin protein levels in MDA-MB-231 cell lines. MDA-MB-231 cells were transfected with either a non-targeting shRNA construct (NTC) or an shRNA targeting vimentin, followed by rescue of vimentin expression with either wild-type (WT) or mutant vimentin constructs. (**B**) A line map is presented to illustrate the overall logical flow and key steps of this study, summarizing how each experiment and result contributes to the overarching conclusions.

Supplementary Videos

Supplementary Video S1 Mitotic RPE1 cells treated with DMSO.

Supplementary Video S2 Mitotic RPE1 cells treated with vinblastine and hesperidin.

Supplementary Video S3 Mitotic MDA-MB-231 cells treated with DMSO.

Supplementary Video S4 Mitotic MDA-MB-231 cells treated with vinblastine and hesperidin.
